# Association between Health Systems Performance and Treatment Outcomes in Patients Co-Infected with MDR-TB and HIV in KwaZulu-Natal, South Africa: Implications for TB Programmes

**DOI:** 10.1371/journal.pone.0094016

**Published:** 2014-04-09

**Authors:** Marian Loveday, Nesri Padayatchi, Kristina Wallengren, Jacquelin Roberts, James C. M. Brust, Jacqueline Ngozo, Iqbal Master, Anna Voce

**Affiliations:** 1 Health Systems Research Unit, South African Medical Research Council, Cape Town, South Africa; 2 Centre for the AIDS Programme of Research in South Africa (CAPRISA), University of KwaZulu-Natal, Durban, South Africa; 3 Tuberculosis & HIV Investigative Network of KwaZulu-Natal (THINK), Durban, South Africa; 4 Retired from Centres for Disease Control and Prevention, Atlanta, Georgia, United States of America; 5 Department of Medicine, Montefiore Medical Center & Albert Einstein College of Medicine, Bronx, New York, United States of America; 6 KwaZulu-Natal Department of Health, Pietermaritzburg, South Africa; 7 King Dinuzulu Hospital Complex, KwaZulu-Natal Department of Health, Durban, South Africa; 8 Discipline of Public Health Medicine, University of KwaZulu-Natal, Durban, South Africa; Public Health Agency of Barcelona, Spain

## Abstract

**Objective:**

To improve the treatment of MDR-TB and HIV co-infected patients, we investigated the relationship between health system performance and patient treatment outcomes at 4 decentralised MDR-TB sites.

**Methods:**

In this mixed methods case study which included prospective comparative data, we measured health system performance using a framework of domains comprising key health service components. Using Pearson Product Moment Correlation coefficients we quantified the direction and magnitude of the association between health system performance and MDR-TB treatment outcomes. Qualitative data from participant observation and interviews analysed using systematic text condensation (STC) complemented our quantitative findings.

**Findings:**

We found significant differences in treatment outcomes across the sites with successful outcomes varying from 72% at Site 1 to 52% at Site 4 (p<0.01). Health systems performance scores also varied considerably across the sites. Our findings suggest there is a correlation between treatment outcomes and overall health system performance which is significant (r = 0.99, p<0.01), with Site 1 having the highest number of successful treatment outcomes and the highest health system performance. Although the ‘integration’ domain, which measured integration of MDR-TB services into existing services appeared to have the strongest association with successful treatment outcomes (r = 0.99, p<0.01), qualitative data indicated that the ‘context’ domain influenced the other domains.

**Conclusion:**

We suggest that there is an association between treatment outcomes and health system performance. The chance of treatment success is greater if decentralised MDR-TB services are integrated into existing services. To optimise successful treatment outcomes, regular monitoring and support are needed at a district, facility and individual level to ensure the local context is supportive of new programmes and implementation is according to guidelines.

## Introduction

In KwaZulu-Natal many patients have multidrug-resistant tuberculosis (MDR-TB), defined as TB resistant to isoniazid and rifampicin. [1] Fuelled by concomitant hyper-endemic TB and HIV, KwaZulu-Natal has one of the largest drug-resistant TB epidemics in the world. [1], [2] Management of MDR-TB is complex, and different interlinked health service components influence each other affecting MDR-TB treatment outcomes. To provide treatment for patients closer to their homes, a decentralised model of treatment was initiated at four sites in 2009. To guide implementation of the MDR-TB programme in the four sites, provincial treatment guidelines were developed and distributed.

Successful implementation of any MDR-TB programme is dependent on different health system components functioning together to support effective service delivery, but there is limited evidence on how programmes interact with health systems and which factors enable or hinder this interaction. [3]–[5] Furthermore, although much has been written about the patient and disease characteristics that impact on MDR-TB treatment outcomes (TOs), there is little evidence of the impact of health system performance on TOs. [6] Negative health care worker attitudes, drug stock-outs, large cohort size and limited resources with which to trace defaulters are some of the factors which have been described as contributing to poor TOs [7]–[10].

To evaluate implementation of the decentralised MDR-TB programme we undertook a study comparing the effectiveness of decentralised care for MDR-TB patients with care in a centralised setting. [11], [12] During the comparison we noted that the MDR-TB programme was implemented differently at each decentralised site. Furthermore, treatment outcomes varied considerably between the sites, with some sites performing better than the centralised hospital and others worse. To better understand the diverse implementation and the subsequent varying treatment outcomes, we performed an analysis of health systems factors. We hypothesized, that treatment outcome was associated with local health system factors and that by investigating the association, we could identify those factors critical to successful treatment outcomes.

To determine that association, “health system performance” (HSP) was defined as one variable. For each site, HSP was the result of a composite assessment of four performance domains: context, integration, mechanism and output. [13], [14] Treatment outcome (TO) at each site, our second variable, was based on the site’s overall treatment outcomes.

In this exploratory prospective mixed methods health systems comparative case study of the four sites, we addressed the following research questions. (1) Is there an association between treatment outcome (TO) and health system performance (HSP)? (2) Which domains of health system performance are associated with successful TOs?

## Methods

### Ethics Statement

The study protocol was approved by the University of KwaZulu-Natal Biomedical Research Ethics Committee (Ref: BF052/09), and by the KwaZulu-Natal Department of Health. Only secondary data, the data routinely collected by health workers for clinical care was used in this study. To protect patient confidentiality and anonymity the data bases were de-identified and access strictly limited. Informed consent was waived by the ethics committee, since all patient data used were previously collected during the course of routine medical care and did not pose any additional risks to the patients.

### Study Design

This mixed methods case study of four decentralized MDR-TB sites between 1 July 2008 and 30 June 2012 was a prospective health systems study.

### Study Population

The Provincial TB directorate identified four sites, the cases for our case study, for implementation of the decentralised MDR-TB programme. These sites were purposively selected in areas where large numbers of patients with MDR-TB were being diagnosed. Although distributed widely across the province the infrastructure and socio-economic status of the populations in these districts was similar. These sites together with their health care workers and managers were included in the study, as were district-level managers involved in MDR-TB management from the districts where the sites were based.

All patients from the four decentralised sites with a culture confirmed diagnosis of MDR-TB, age ⩾18 years, and who commenced treatment between I July 2008 and 30 June 2010, were included in the study. No data was collected after October 1, 2012. Inclusion criteria for the comparison study required that patients reside within the catchment area of the site. Patients receiving care at more than one site were excluded, as were patients who had MDR-TB with additional resistance to amikacin, kanamycin, capreomycin or any fluoroquinolone.

### Data Collection

We reviewed medical records to collect patient-related demographic, clinical, pharmaceutical and laboratory data. All data, was collected prospectively, prior to knowledge of patient treatment outcomes. Health system data was collected from different components of the health system - laboratory, pharmaceutical and transport services and human resources - using existing records and databases, structured questionnaires, observation and interviews. As differences between the sites and complexities emerged, an iterative approach enabled us to identify new health system data required and develop appropriate data collection methodologies.

Quantitative data was complemented by qualitative data obtained through participant observation and discussions with staff. Over the four year study period each site was visited monthly for a day (ML). During each visit data from each health system component was collected (see Table S1), the functioning of the MDR-TB unit observed and informal discussions held with the nurse-in-charge of the MDR-TB unit, the clinician responsible for MDR-TB and the hospital pharmacist. Through a process of on-going reflection, feedback and discussion with facility and district level staff problems were investigated to determine their origin and cause and possible solutions identified. Field notes detailing the visit and documenting observations and discussions with staff were written up after returning from the site. Notes were also made of concerns, opinions and issues which needed follow up.

### Variable Definitions

In defining the HSP variable we adapted a conceptual framework, which had been validated both internationally and in our setting, [13], [14] and identified key domains of health system performance – context, integration, mechanism (comprised of support services and human resources) and output (Table 1). To measure HSP we identified health system factors which would affect system performance. Concomitant indicators with which to measure the impact of these factors on each domain were then identified and defined (Table 1).

**Table 1:** Framework to monitor health system factors at 4 decentralised MDR-TB sites.

Sub-domains	Indicators measuring local site health system factors
Health system factors	
**Domain: Context**	
District level: Leadership and ownership	MDR-TB perceived as a district problem and not as an MDR-TB unit problem.
	District prioritises spending on MDR-TB programme.
District level support: Managerial, administrative, technical	Staff at PHC sites adequately trained to manage down-referred MDR-TB patients.
	Regular visits by district TB co-ordinator.
Facility level support	Staff at MDR-TB unit feel supported by facility managers.
**Domain: Integration**	
Integrated services: MDR-TB and HIV	Integrated services
	Integrated clinical notes
Integrated services: MDR-TB and PHC	Mobile clinics re-organised to ensure tracing of TB/MDR-TB defaulters and injecting MDR-TB patients at home.
Integrated services: MDR-TB and TB	Communication system for discussing and solving problems with down-referral.
**Domain: Mechanism**	
Human Resources (HR)	Availability of staff
	Knowledge
	Stability and consistency (including staff rotation)
	Managerial support
Support services (SS)	Pharmaceutical: Availability of drugs
	Laboratory: Culture turnaround time
	Transport: Needs satisfaction – Percentage of transport requests met
	Equipment: Availability, functionality and utilisation
**Domain: Output**	
Continuity of care	Referral system: Treatment initiation delay
	Mechanism for following up defaulters
	Monitoring and evaluation system in place: MDR-TB register up to date
Quality of care	Availability of clinical guidelines
	Adherence to guidelines: Audit of clinic notes (clinical skills)
	Clinical notes adequate and complete: Audit of clinic notes (clinical skills)
	Utilisation of clinical expertise at centralised, specialised hospital: Audit telephone calls to doctors at centralised hospital
	Management of serious adverse events immediate and appropriate

To measure indicators, data collected at each site was scored by an investigator (ML) and the nurse-in-charge of the MDR-TB unit (Table 2). Where appropriate, the score incorporated the date of implementation (earlier was scored higher than later) and consistency (greater consistency scored higher than partial or lower consistency). A total score for each domain was calculated and converted where necessary, so that each domain was equally weighted. The sum of the domain scores provided an overall HSP score for each site with the maximum possible score for any one site being 160. Annually, over the four year study period a HSP score per site was calculated. At the end of the study an average score for each indicator, health system factor and domain was calculated for each site (Table 3). Similar scoring methodologies have been used to measure TB and HIV integration at primary level clinics in South Africa. [15], [16].

**Table 2:** Examples of Indicators with measurement and scoring systems.

Criteria for measurement/Indicators	Evidence	Scoring system
**Health system factors: District level: Leadership and ownership**		
MDR-TB perceived as a district programme and not as an MDR-TB unit programme.	Documented evidence:Minutes of quarterly	Yes or noDate this started
MDR-TB reported and discussed in quarterly district TB meetings	district TB meetings	Consistency
**Integrated services: MDR-TB+HIV**		
% TB and HIV co-infected patients receive MDR-TB/HIV consultationand management at one desk	Observation	Yes or noDate this started
		Consistency
% co-infected patients who do not queue at pharmacy	Observation	Yes or noDate this started
		Consistency
% clinical notes of co-infected patients which on discharge detail referral for ART	Audit of clinic notes	Yes or noDate this started
		Consistency
**Integrated MDR-TB and PHC services**		
% mobile clinics re-organised to ensure tracing of TB/MDR-TB defaulters andinjecting MDR-TB patients at home	Transport audit	No. of vehicles Date this started
	Vehicle logs	Consistency
**Continuity of care**		
Mechanism for following up defaulters: % patients who miss visits who arefollowed up and his is documented in folder	Audit of clinic notes	Yes or no

**Table 3:** Scores allocated for health system factors at the 4 decentralised sites.

Health system factor	Indicator	Maximum scorepossible	Site1	Site2	Site3	Site4
**Domain: Context**						
District level: Leadership and	MDR-TB perceived as a district problem and notas an MDR-TB unit problem	8	8	0	0	3
ownership	District prioritises spending on MDR-TB programme	5	3	1	1	1
District level support:Managerial, technical	Staff at PHC sites adequately trained to manageMDR-TB down-referred patients	8	6	3	2	3
+ administrative	Regular visits by district TB co-ordinator	5	2	0	0	1
Facility level support	MDR-TB unit staff feel supported by facility managers	3	2	0	0	0
	Total context score	29	21	4	3	8
**Weighted context score**		**40**	**29**	**6**	**4**	**11**
**Domain: Integration**						
**Integrated MDR-TB and HIV**	Integrated services	13	13	9	7	6
	Integrated clinical notes	8	8	5	5	3
Integrated MDR-TB and PHC	Mobile clinics re-organised to ensure tracing ofTB/MDR-TBdefaulters and injecting MDR-TB patients at home.	8	8	5	3	0
Integrated MDR-TB and TB	Communication system for discussing andsolving problems with down-referral	3	3	2	0	3
	Total integration score	32	32	21	15	12
**Weighted integration score**		**40**	**40**	**24**	**19**	**15**
**Domain: Mechanism**						
Human resources	Availability of staff	10	2	7	7	5
	Knowledge	3	3	3	1	3
	Stability and consistency	4	4	2	2	2
	Managerial support	4	4	3	0	0
Support services	Pharmaceutical: Availability of drugs	4	3	0	3	2
	Laboratory: Culture turnaround time	8	8	8	5	1
	Transport: Needs satisfaction - % of transport requests met	1	1	0	1	0
	Equipment: Availability and utilisation	5	5	3	1	1
	Total mechanism score	39	30	26	20	14
**Weighted mechanism score**		**40**	**31**	**27**	**20**	**14**
**Domain: Output**						
Continuity of care	Referral system: Treatment initiation delay	9	4	4	4	2
	Mechanism for following up defaulters	3	3	3	2	3
	M+E system in place: MDR register up to date	3	3	2	2	2
Quality of care	Availability of clinical guidelines	5	5	1	3	2
	Adherence to guidelines: Audit of clinic notes	3	1	0	0	0
	Clinical notes adequate and complete: Audit of clinic notes	3	3	2	2	1
	Utilisation of clinical expertise at KGV:Audit telephone calls to KGV doctors	3	3	1	2	1
	Management of serious adverse events immediate and appropriate	6	6	6	6	6
	Total output score	35	28	19	21	17
**Weighted output score**		**40**	**32**	**22**	**24**	**19**

Treatment outcomes (TOs) of patients were determined at the end of treatment, according to definitions developed by the WHO (Table 4), [17], [18] based on patient data indicating successful treatment (patient was cured or completed treatment) or unsuccessful treatment (failure to respond to treatment, default or death). The overall percentage of these outcomes was calculated for each of the four sites.

**Table 4:** Treatment outcome definitions*.

Treatment outcome	Definitions
Cure	Cure was defined as completion of treatment and ≥5 consecutivenegative culture results in the final 12 months of treatment.
Treatment completion	Treatment completion referred to completion of therapy but withoutbacteriologic documentation of cure.
Treatment success	Treatment success has been defined as the percentage of patients in whom the treatment outcomewas either cured or completed. That is, “% successful = no. of patientscured+no. of patients completed treatment/Total no. initiated treatment×100”.
Treatment failure	Treatment failure was defined as having more than one positive culture in the final 12 months of therapy,or if any one of the final three cultures was positive, or if more than one drug in the treatment regimen was replaced,or if treatment was terminated due to adverse events or no clinical improvement.
Default	Default was defined as an interruption in treatment for ≥2 consecutive months for any reason.
Death	Death was defined as all-cause mortality during MDR-TB treatment.
Unsuccessful treatment	Unsuccessful treatment outcome has been defined as the percentage of patients in whom the treatmentoutcome was died, defaulted, or failed treatment.
Transferred out	Transferred out: A patient with MDR-TB who was transferred to another reporting andrecording unit a year after study-enrolment whose treatment outcome is unknown.

*Treatment outcome definitions used are WHO definitions for the management of MDR-TB. [17], [18]

### Analysis

Data analyses were conducted using SAS version 9.2 (SAS Institute Inc., Cary, NC, USA). Differences in outcomes across sites were compared using binomial regression. Statistical significance was set at alpha = 0.05. Pearson Product Moment Correlation coefficients were used to quantify the direction and magnitude of association between HSP scores and successful TO by domain.

Qualitative data was explored and analysed in light of the introduction of a new programme. Using systematic text condensation (STC) the field notes were read and possible themes identified. [19] Through decontextualisation and a process of reflection on commonalities and differences, themes were classified as codes. Condensation provided meaning to the codes which were finally synthesised into our conceptual domains.

## Results

### Treatment Outcomes and HSP Scores

TOs of the 736 patients treated at the 4 decentralised sites are tabulated in Table 5. Across the four sites, 76% of all patients were co-infected with HIV. Overall, 58% of patients at the decentralised sites had successful TOs (cured and/or completed). However, there were differences in treatment outcomes across the sites. Successful TOs varied from a high of 72% at Site 1 to a low of 51.7% at Site 4 (p<0.01) and Site 3 and Site 4 had significantly higher default and death rates respectively (p<0.01). A detailed comparison of patient characteristics and TOs has been reported. [12].

**Table 5:** Treatment outcomes of patients with MDR-TB treated at 4 decentralised sites in KwaZulu-Natal, South Africa*.

Treatment Outcomes	Site 1	Site 2	Site 3	Site 4	*p*-value	All decentralized hospitals
	n = 125	n = 148	n = 202	n = 261		n = 736
Treatment success	90 (72.0)	89 (60.1)	113 (55.9)	135 (51.7)	<0.01	427 (58.0)
Died	17 (13.6)	22 (14.9)	25 (12.4)	69 (26.4)	<0.01	133 (18.1)
Failed	7 (5.6)	11 (7.4)	12 (5.9)	19 (7.3)	0.87	49 (6.7)
Defaulted	9 (7.2)	20 (13.5)	50 (24.8)	28 (10.7)	<0.01	107 (14.5)

Data are number (%), unless otherwise indicated.

*Treatment outcome definitions used are WHO definitions, as defined in Table 4.

**Figure 1:**
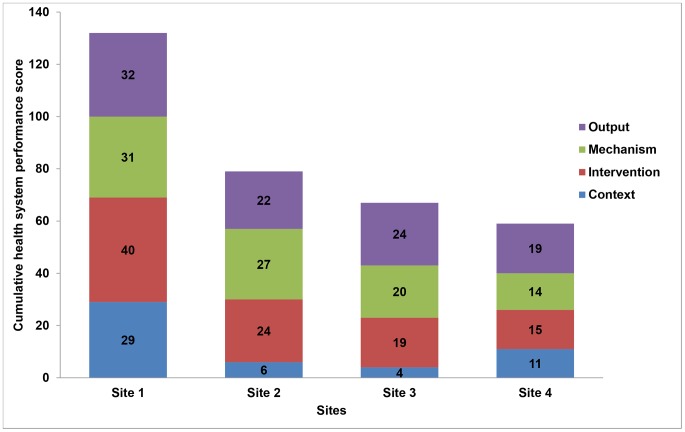
Breakdown of total health systems performance score by domain at 4 decentralised MDR-TB sites. The four sites are plotted on the X-axis and the health system performance score on the Y-axis. Health system performance is the sum (cumulative score) of the four different domains (output, mechanism, integration and context), which are shaded differently. Site 1 had the highest score of 132 which comprised scores of 32 for the output domain, 31 for the mechanism domain, 40 for the integration domain and 29 for the context domain.

Overall HSP scores varied across the sites with Site 1 having the highest HSP score (132 out of a possible 160) and Site 4 the lowest score (59 out of a possible 160) (Figure 1). Site 1 scored highest score in all domains, achieving the maximum score of 40 in the domain ‘integration’ compared to Site 2 (score = 24), Site 3 (score = 19), and Site 4 (score = 15). In contrast, Site 4 scored the lowest in three of the four domains with ‘context’ being the sole exception.

### Analysis by Domains

Context domain scores varied from a high of 29 at Site 1 to a low of 4 at Site 3 (Figure 1). As a consequence of regular on-site support visits by the district TB coordinator staff and facility manager support, staff at Site 1 felt supported by the district and local hospital, in contrast to staff at the other sites (Table 3: Context domain: District and facility level support).

*‘Whenever we have a problem we phone the district TB co-ordinator. She is strict with us, but is also helpful.’*

(Interview: Site 1: Nurse-in-charge of MDR-TB outpatients clinic)

*‘The district TB co-ordinator came to the opening of this MDR-TB unit……But since then has never been near. Him and the hospital managers…….they don’t even know where the unit is.’*

*‘The hospital managers help with sorting out problems? Never, not one!’*

(Interview: Site 3: Nurse-in-charge of MDR-TB outpatients clinic)

Integration domain scores varied from a high of 32 at Site 1 to a low of 12 at Site 4 (Figure 1). The two observations below are examples of the effect of the context domain on integration (Table 3: Integration domain: Integrated MDR-TB and PHC).

*Additional mobile clinics for the MDR-TB programme were identified by the district in the first year of the programme (2008). The district mobile services were re-organised so that each mobile provided services for all TB, HIV and MDR-TB patients in a smaller geographical area than that in which mobiles had previously operated attending to TB and HIV patients.*

(Field notes Site 1: Repeated observations (ML) 2009–2012)

*‘I still don’t have a mobile vehicle for MDR-TB. The district just ignores me and the hospital managers aren’t concerned.’*

(Interview with the nurse-in-charge of MDR-TB unit at Site 3, 12 November 2011. Two mobile vehicles were made available in January 2012, almost 4 years after the start of the MDR-TB programme.)

From the mechanism domain a number of human resource issues affecting the functioning of sites 2–4 emerged. The stability of the health services was affected by the common hospital practice of the rotation of front-line health workers through the different clinical disciplines – a regular practice at sites 2 and 3. At these sites key clinical staff were rotated every three months through the different clinical disciplines, including the MDR-TB unit, leading to low scores for knowledge about MDR-TB, and poor stability and consistency in the services (Table 3). At Site 1, as two staff members were on extended sick leave, the site had a low score for availability of staff. However, this was offset by other HR factors - the stability and knowledge of remaining staff together with support for these staff.

Key implementation posts were filled by the same staff members from 2008–2012. *The nurse-in-charge of the MDR-TB outpatients knows all the patients and as soon as they miss a monthly appointment are phoned and encouraged to come back.*

(Field notes Site 1: Repeated observations (ML) 2009–2012)

The nurse-in-charge of the MDR-TB outpatients was rotated every three months. *The appointment systems were not functional, as she didn’t know the patients and was unaware if they missed appointments. Consequently, this site had a significantly higher default rate than the other sites (p<0.01) (* *Table 3* *).*

(Field notes Site 3: Repeated observations (ML) 2009–2012: Interpretation of TOs: August)

Consistency of services was undermined by a second common human resource practice - the appointment of managers in ‘acting’ positions. Site 4 scored a 0 for managerial support (Table 3).

I took up a problem that had emerged with the ‘acting’ head of Site 4 MDR-TB unit.

*‘Well, I’m only acting. I can’t do anything.’*

(Field notes and interview September 2010: Acting head of the Site 4 MDR-TB unit)

From the output domain quality of care varied across the four sites and inadequate clinical skills together, and poor adherence to clinical guidelines contributed to fewer successful TOs at Sites 2, 3 and 4 (Table 3).

*‘I am only working in the MDR-TB unit for 3 months. I haven’t seen any guidelines. I follow what was done before. Dr. X who worked here before is around. I haven’t spoken to him. He is busy, and so am I.’* (Interview with clinician at Site 2, April 2010)

*Doctors at this site are rotated through the MDR-TB unit every three months and not all of them familiarise themselves with the guidelines. A new doctor stopped the injectable phase in three patients after four months, two months too early. Two of the patients subsequently failed treatment.*

(Observational data at Site 2: Repeated observations (ML) 2009–2012: Interpretation of TOs August 2012)

### Association between TOs and HSP

**Figure 2:**
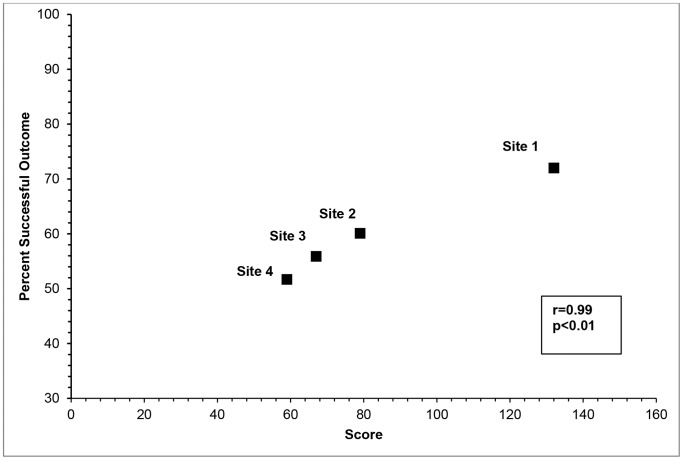
Association between successful treatment outcomes and total health systems performance score at 4 decentralised MDR-TB sites. This figure shows the association between successful treatment outcomes and total health systems performance score. The percentage of successful treatment outcomes is plotted on the Y-axis and the health performance score on the X-axis. From the graph it can be seen that Site 1 had the highest treatment success and highest total health system performance score. Sites 2, 3 and 4 can be seen to have lower health system performance scores and lower rates of treatment success. This graph shows there was an association between successful treatment outcomes and total Health System Performance score (r = 0.99) and that this association was significant (p<0.01).

We found a correlation between successful TOs and total HSP score (r = 0.99, p<0.01) (Table 6, Figure 2). Quantitative data analysis suggested that the domain ‘integration’, had the strongest association with successful treatment outcomes (r = 0.99, p<0.01) (Table 6). However, qualitative data indicated that issues within the domain ‘context’ influenced both programme implementation and the ‘integration’ domain.

**Table 6:** Correlation between health system performance and successful treatment outcomes for each domain for MDR-TB patients treated at 4 decentralised sites.

	Pearson Correlation Coefficient, r	p-value
Total	0.99	0.01
Context	0.82	0.18
Integration	0.99	<0.01
Output	0.94	0.06
Mechanism	0.93	0.07
Human resources (HR)	0.42	0.58
Support services (SS)	0.96	0.04

## Discussion

Our exploratory study suggests there is an association between treatment outcomes (TOs) and Health System Performance (HSP). We found this association to be significant (r = 0.99, p<0.01), with Site 1 having the highest number of successful TOs and the highest total HSP score. Conversely, Site 4 had the lowest number of successful TOs and the lowest HSP score. In addition, our study suggests that the ‘context’ and ‘integration’ domains had the strongest association with successful TOs.

District level ownership and leadership enabled re-organisation and realignment of services at Site 1, the ‘integration’ domain. Different health system components, such as pharmaceutical and transport services, were incorporated into the new programme and, the inclusion of local key personnel with grounded clinical experience and knowledge of the local situation resulted in the development of a model of care which built on available strengths and was appropriate for local needs. [20]–[22] For front line health workers and facility managers at Site 1, district-level ownership translated into regular on-site support visits by the district TB co-ordinator. These visits provided encouragement and supervision, and aided in the resolution of site-level problems, thereby enabling health care workers to deal with emerging difficulties instead of becoming ‘mired in inertia’. [23] In addition, the visits led to increased accountability and a commitment to patient care resulting in improved adherence and a higher number of successful TOs.

In contrast to Site 1, district and facility managers at Sites 2, 3 and 4 in failing to own the MDR-TB problem, underestimated the realignments and changes necessary for the health system. This led to partial implementation of the programme, limited managerial and support services support, delays in the re-organisation of the human resources and re-allocation of vehicles and poor integration of the MDR-TB programme into existing PHC services. Furthermore, as staff at Sites 2, 3 and 4 were not visited regularly they felt unsupported and unvalued, were unmotivated, and lacked commitment to their work and their patients, resulting in fewer successful TOs. Other authors support this interpretation; they have shown that ‘context’ has a high impact on the capacity of health services to co-ordinate and support effective service delivery. [24]–[26] In a study evaluating the HIV programme in Russia, Tkatchenko-Schmidt et al [27] described the impact of leadership, ownership and support on staff motivation and performance. A systematic review which examined the relationship between nursing leadership and patient outcomes found a significant association between positive leadership and increased patient satisfaction and reduced adverse events. [28] Other studies have documented the effect of political and managerial leadership on health system functioning and the increased likelihood of unsuccessful TOs when health systems are dysfunctional.[26], [29]–[32].

Much has been written about the need for and importance of vertical health programmes strengthening health systems. [33], [34] In our study, district level leadership at Site 1 integrated the new decentralised MDR-TB vertical programme into the existing district health services. The re-alignment and re-organisation of the services enabled the district health system to benefit from and be strengthened by the introduction of a new programme. In contrast, at the other sites, the new programme with its additional resources were not integrated into horizontal service delivery, and the existing services neither strengthened nor capacitated. In addition, the integration of the MDR-TB and HIV programmes at Site 1 would have contributed to successful TOs, as the role of ART in the successful treatment of co-infected patients is well documented. [35], [36].

Inadequate clinical skills together with delayed implementation of changes to clinical guidelines contributed to Sites 2, 3 and 4 scoring poorly in the quality of care component of the domain ‘output’ and achieving fewer successful TOs. In a recent article on new TB diagnostics, the difficulties in implementing new guidelines or changes to guidelines are described. [37] In this article the authors emphasise that even small changes in guidelines or algorithms are a major undertaking in a national TB programme. Given recent advances in TB diagnostics and therapy and possible changes to algorithms and regimens, national TB programmes need to be cognisant of the complexity of change. Up to date guidelines must be available at facilities and in our study guidelines were consistently available at Site 1 only (Table 3). Other studies have documented the negative impact of unavailable guidelines and protocols on quality of care. [38], [39].

To ensure that the most up-to-date guidelines and regimens are implemented, regular on-going training, support and supervision at the decentralised sites is necessary. Given that the sites are scattered throughout a large province, different models for providing ongoing training, such as electronic or in-service, need to be explored. [40], [41] In addition, regular, careful support and supervision improves quality of services significantly. [42] If staff feel supported and confident, they are less likely to seek alternative employment. Moreover, to ensure optimal implementation, district and facility managers need support, supervision and monitoring to ensure they take ownership of a new programme, take responsibility for service re-organisation and re-alignment and provide support.

Besides the lack of support for front-line staff at Sites 2, 3 and 4, two routine HR practices contributed to poor HSP. This regular rotation of clinical staff contributed to the loss of valuable skills and experience and instability and a lack of continuity in MDR-TB management. The practice of rotation has to be reconsidered for key clinical positions such as the doctor responsible for MDR-TB services and the nurse-in-charge of the outpatient clinic. If rotation is necessary, a longer rotational cycle would reduce the rapidity with which skills are lost.

Secondly, in resource-constrained settings, a strategy used to reduce personnel costs is to appoint staff as ‘acting’ managers. Site 4 had an acting facility manager for much of the study period, which, together with a number of other factors contributed to the poor HSP at this site. This stalling tactic for key delivery positions is counter-productive resulting in unmotivated and unproductive staff, poor service implementation and poor patient care.

The importance of the head of the outpatient clinic in health services for chronic and long-term conditions is seldom recognised. In decentralised MDR-TB services, this person is the interface between MDR-TB and PHC services and the community as well as being the gateway to tertiary care. First, she has to ensure that the service is patient-focussed, the patient is supported and constant education is provided to promote adherence. Second, she is responsible for co-ordination of two different programmes (TB and HIV), and for the organisation of different and diverse support service components to ensure health service performance is optimal. And third, she acts as a liaison between the different levels of care so that the patient always receives appropriate treatment.

### Limitations

This study was an evaluation of an intervention implemented in the public sector, providing evidence under routine conditions which supports wider applicability of results. However, it was subject to challenges experienced in this sector. Our data used for the evaluation was the data routinely collected by health workers, which at times, was incomplete and inaccurate. Although we collected individual patient data, the routinely collected health system data were not available at an individual level. And, therefore, we were unable to determine the impact of HSP on individual patient TOs. By focusing on health system performance other factors, such as differences in baseline characteristics, which may have influenced TOs were not included in the analysis. Furthermore, the small sample size (4 sites) lessened the power of tests, reduced precision, increased the effect of variability and precluded the use of more sophisticated methods to determine the association of different domains to one another.

## Conclusions

This study is a first step toward predicting which health system factors affect treatment outcomes (TOs). In spite of the above limitations, we have shown that there is a trend between HSP and TO and a larger case control or comparative study is warranted. To conclude, we suggest that decentralised MDR-TB management can improve treatment outcomes if district leadership is effective, management takes ownership of the problem and provides support by re-organising and re-aligning health service components, allocating sufficient financial resources, and providing regular visits and assistance in resolving emerging problems. Moreover, a vertical programme can strengthen district level health systems if it is integrated into existing services.

As MDR-TB prevalence increases, health services expand, and different models of care are introduced, we recommend regular monitoring and support of district and facility managers and individual health workers to encourage service integration, guideline adherence and optimize TOs.

In addition, we have identified HR practices that are detrimental to HSP: rotation of staff in key clinical positions and the appointment of managerial staff in an ‘acting’ capacity. We recommend alternatives to these practices.

## Supporting Information

Table S1 (XLS)
